# *RSC Advances* Outstanding Student Paper Awards 2023

**DOI:** 10.1039/d4ra90089h

**Published:** 2024-09-11

**Authors:** Laura Fisher

## Abstract

We are delighted to announce the winners of the *RSC Advances* Outstanding Student Paper Awards 2023. These awards recognise outstanding work published in the journal, in which a substantial component of the research was conducted by a student.
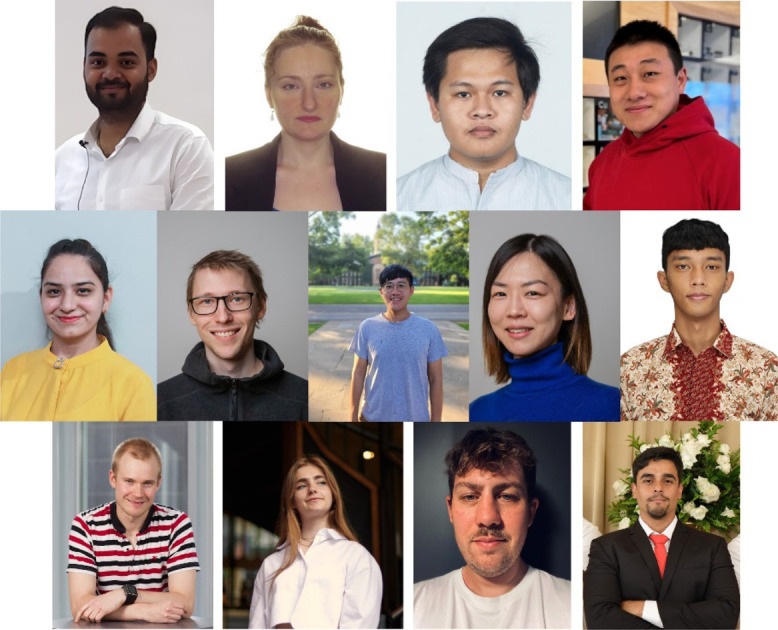

We are delighted to announce the winners for the *RSC Advances* Outstanding Student Paper Awards 2023. *RSC Advances* presents an annual award series to recognise the hard work of students within the chemistry community.

All research articles published in *RSC Advances* in 2023 were considered. In order to be eligible for this award, the first author or co-first author must have been a student at the time of carrying out the research. From the support of corresponding authors, we received over 700 nominations highlighting the incredible talent and potential within the next generation of chemists. It is particularly inspiring to learn about the exceptional work from a diverse range of research fields and countries, a testament to the quality of research and curiosity throughout the community.

The nominations were shortlisted based on a number of criteria, and the winning papers were then selected by our Editorial Board and Associate Editors (Table 1).

**Table 1:** Winners of the Outstanding Student Paper Awards 2023

*Analytical chemistry*	Kumar Shwetabh, Indian Institute of Technology (Indian School of Mines), India
*Biological and Medicinal Chemistry*	Mateusz Kozarski, University of Warsaw, Poland
*Catalysis*	Respati K. Pramadewandaru, University of Ulsan, Republic of Korea
*Computational & Theoretical Chemistry*	Songyuan Yao, University of Oklahoma, USA
*Energy Chemistry*	Naufal Hanif Hawari, A*STAR (Agency for Science, Technology and Research), Singapore
*Environmental chemistry*	Valtteri Suorsa, Miho Otaki and Topi Suominen, University of Helsinki, Finland
*Inorganic Chemistry*	Anderson Moledo Vicente Guedes, Federal University of Rio de Janeiro, Brazil
*Materials Chemistry*	Kun-Lin Wu, University of Washington, USA
*Nanoscience*	Ashima Makhija, Maharshi Dayanand University, India
*Organic Chemistry*	Margarita Damai, London Metropolitan University, UK
*Physical Chemistry*	Maria Dekermenjian, INRS-EMT, Canada

Below, we highlight the winner of each subject category, and the research paper that won them the award. Please join us in congratulating all of our winners for their exceptional achievement. We look forward to witnessing their continued growth and impact as they embark on a promising career in the field of chemistry.

## Analytical Chemistry

**Kumar Shwetabh, Indian Institute of Technology (Indian School of Mines), India** 
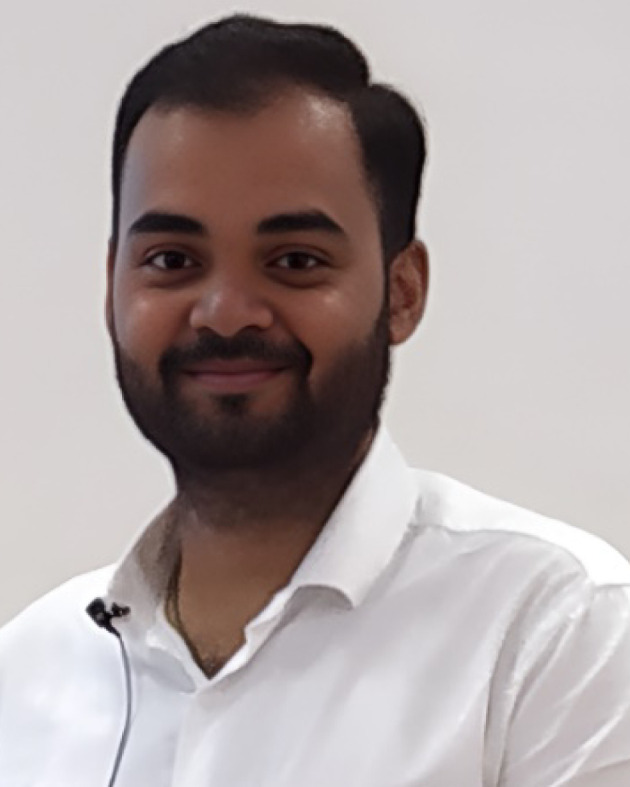


Kumar is recognised for his outstanding contribution to the research presented in:

*Synthesis and upconversion emission studies of CaYF*_*5*_ *:Ho* ^*3+*^ */Yb* ^*3+*^ *phosphor and its applications in optical thermometry, fingerprint detection, and security ink* (https://doi.org/10.1039/D3RA00644A)

Kumar Shwetabh, from Bihar, India, completed his BSc and MSc in physics in 2015 and 2018 respectively, from Lalit Narayan Mithila University, India. He joined the Indian Institute of Technology (Indian School of Mines) for a PhD, and completed his PhD in July 2024 under the supervision of Prof. Kaushal Kumar at the Department of Physics, IIT(ISM) Dhanbad, India. During his PhD, he explored various rare earth-doped nanomaterials for upconversion-based multifunctional applications *via*, biomedical, optical thermometry, forensics, and security applications. His work is mainly focussed on rare earth-doped fluoride-based materials NaYF_4_, NaGdF_4_, LiYF_4_, CaYF_5_, and KYF_4_ nanoparticles. In his spare time he likes to read books and play cricket.

## Biological and Medicinal Chemistry

**Mateusz Kozarski, University of Warsaw, Poland** 
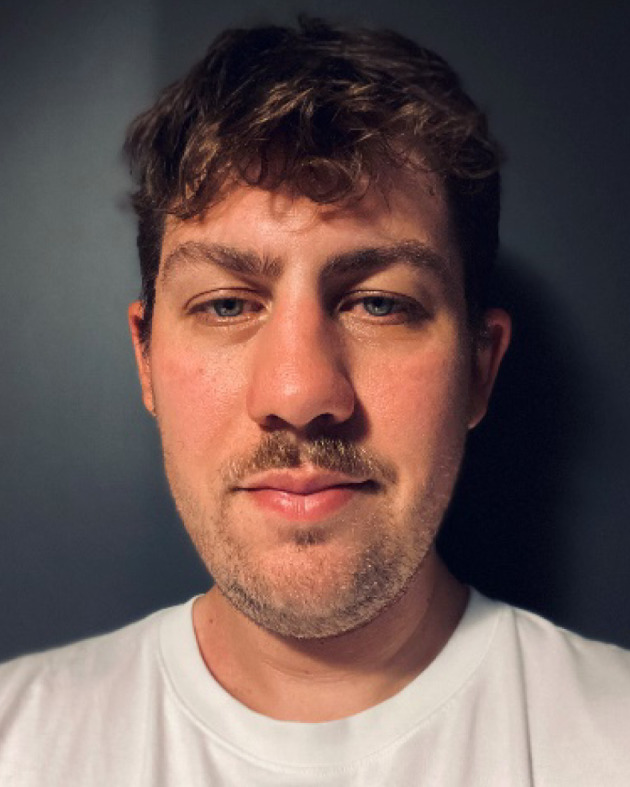


Mateusz is recognised for his outstanding contribution to the research presented in:

*Towards superior mRNA caps accessible by click chemistry: synthesis and translational properties of triazole-bearing oligonucleotide cap analogs* (https://doi.org/10.1039/D3RA00026E**)**

Mateusz Kozarski completed his BSc in molecular biophysics at the University of Warsaw in 2016, focussing on the application of copper(i)-catalysed alkyne–azide cycloaddition to synthesise novel 7-methylguanosine nucleotide analogs modified at the 5′ position. Continuing at the University of Warsaw, he completed his MSc in molecular biophysics in 2018, exploring new molecular tools to investigate the biological role of the enzyme cNIIIB through the design, synthesis, and evaluation of novel 7-methylguanosine 5′-monophosphate analogs.

His pursuit of knowledge led him to a PhD programme at the Faculty of Physics, University of Warsaw, and the Centre of New Technologies, University of Warsaw, in the Chemical Biology and Biophysical Chemistry Laboratory. His doctoral research, supervised by Joanna Kowalska, PhD, DSc, and Prof. Jacek Jemielity, focussed on the synthesis and evaluation of nucleoside-based molecular tools for monitoring mRNA-related biological processes, where he co-authored three scientific publications on 5′-end mRNA metabolism.

From 2022 to 2023, he worked in industry as a scientist at Celon Pharma (Poland), where he was involved in research and development in the mRNA bioengineering group, working on RNA synthesis and purification. Mateusz is currently a Senior Scientist at Etherna (Belgium), a biotechnological company which focuses on customizable Lipid Nano Particles (cLNPs), supported by RNA chemistry and process technologies, here, Mateusz is responsible for optimising RNA production and purification processes.

## Catalysis

**Respati K. Pramadewandaru, University of Ulsan, Republic of Korea** 
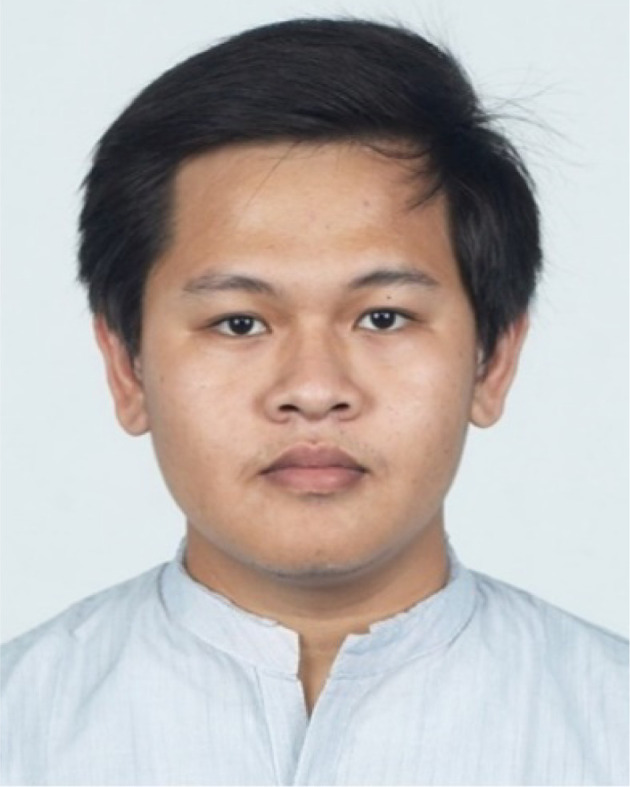


Respati is recognised for his outstanding contribution to the research presented in:

*Synergistic effect of bimetallic Pd–Pt nanocrystals for highly efficient methanol oxidation electrocatalysts* (https://doi.org/10.1039/D3RA04837C)

Respati Kevin Pramadewandaru is an assistant professor in the Department of Materials and Metallurgical Engineering at Sepuluh Nopember Institute of Technology (ITS), Indonesia. He earned his PhD in nano-energy chemistry from the University of Ulsan, South Korea (2024), where he also completed his Master’s degree. His research focuses on material synthesis, characterisation, and applications, particularly in electrochemical systems and nanotechnology.

Respati has published several papers in reputable journals and has received awards such as the Best Paper Presentation at the International Joint Symposium of Ulsan University and Fukuoka University Conferences in 2022. He is actively involved in professional organizations, including the Indonesian Engineers Association (PII), and collaborates with Nanyang Technological University (NTU) on hydrogen energy projects (REIDI ITS) and the fuel cell team with NTU-Univeristas Indonesia (UI).

## Computational & Theoretical Chemistry

**Songyuan Yao, University of Oklahoma, USA** 
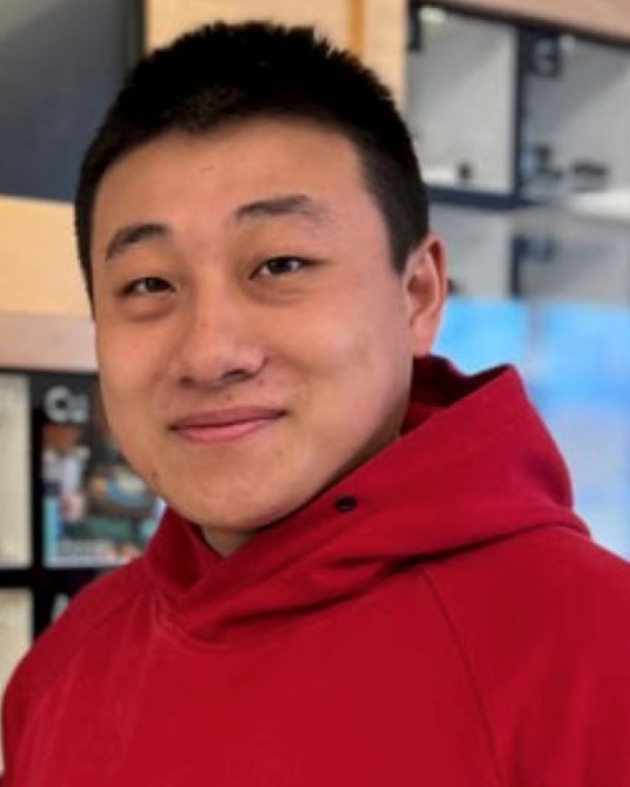


Songyuan is recognised for his outstanding contribution to the research presented in:

*Machine learning based implicit solvent model for aqueous-solution alanine dipeptide molecular dynamics simulations* (https://doi.org/10.1039/D2RA08180F)

Sonyuan Yao graduated with a PhD from Dr Yihan Shan’s group at the University of Oklahoma. His primary research focus during his studies was on utilising machine learning to assist in molecular dynamics simulations. In addition to his work in simulation and dry lab research, he also engaged in wet lab activities, which enhanced my understanding of drug discovery and molecular modelling.

## Energy Chemistry

Naufal Hanif Hawari, A*STAR (Agency for Science, Technology and Research), Singapore 
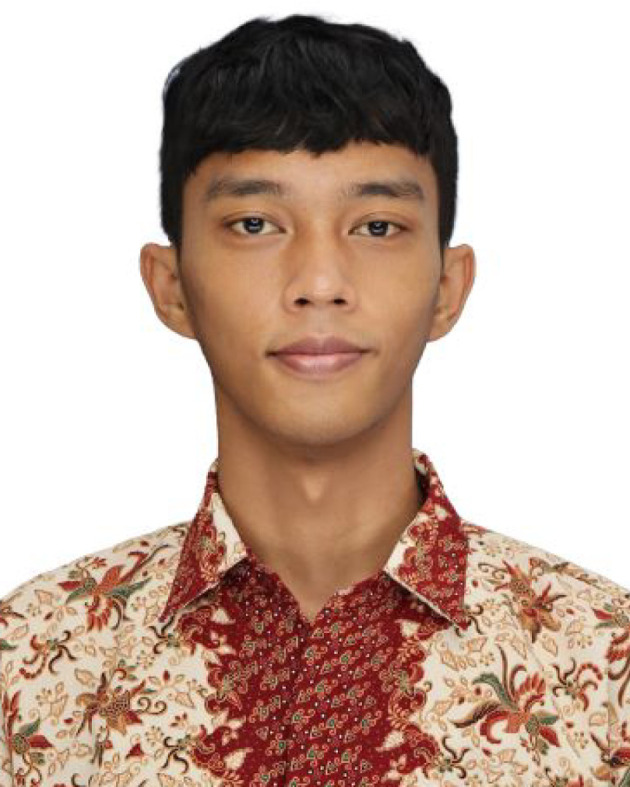


Naufal is recognised for his outstanding contribution to the research presented in:

*Understanding SEI evolution during the cycling test of anode-free lithium-metal batteries with LiDFOB salt* (https://doi.org/10.1039/D3RA03184E)

Naufal Hanif Hawari earned his BSc in 2020 and MSc in 2023, both in materials science and engineering from the Institut Teknologi Bandung, Indonesia. His research focuses on developing high-energy density batteries, including silicon anodes and lithium metal batteries under the supervision of Dr Afriyanti Sumboja. During the final year of his Master’s program, he received the Singapore Pre-Graduate Award (SIPGA), allowing him to spend five months at the Institute of Materials Research and Engineering (IMRE) at the Agency for Science, Technology, and Research (A*STAR) in Singapore. Additionally, he was awarded the Singapore International Graduate Award (SINGA) from A*STAR to pursue a 4 year PhD program at Nanyang Technological University (NTU) under the supervision of Dr Ning Ding and Prof. Yan Qingyu.

## Environmental Chemistry

**Valtteri Suorsa, Miho Otaki and Topi Suominen, University of Helsinki, Finland**

Valtteri, Miho and Topi are recognised for their outstanding contribution to the research presented in:

*Anion exchange on hydrous zirconium oxide materials: application for selective iodate removal* (https://doi.org/10.1039/D2RA06489H)
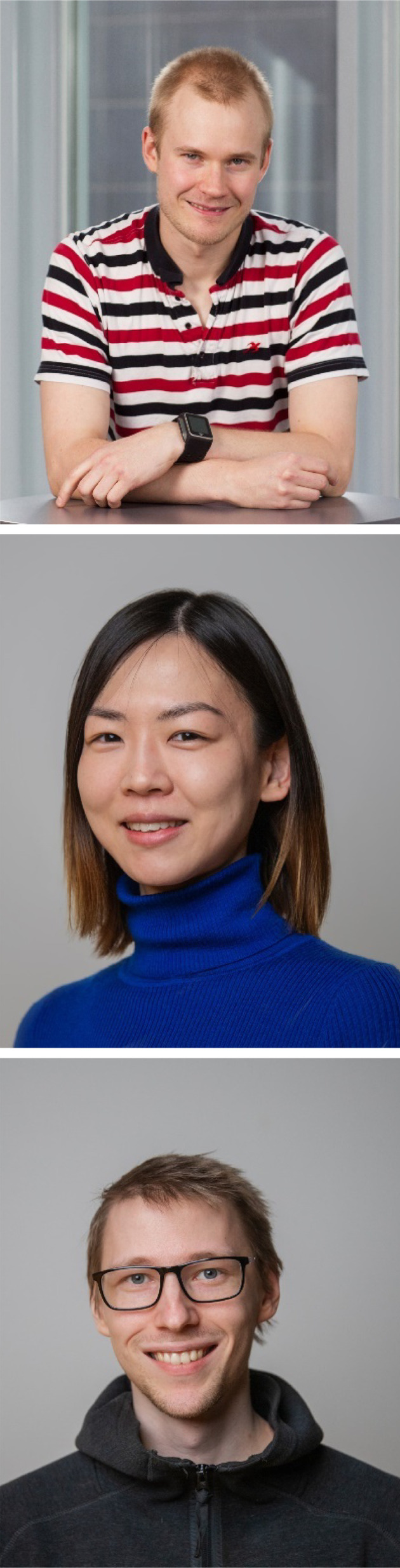


Valtteri Suorsa obtained his PhD in the CHEMS Doctoral School at the University of Helsinki, Finland, in 2023. Within his PhD studies he and his co-author colleagues in Associate Professor Risto Koivula’s ion exchange for nuclear waste treatment and recycling research group, focused on different ion exchange materials suitable for purification of radioactive waste. His doctoral thesis ‘Selective iodate removal using zirconium oxides’ summarises his research regarding the utilization of zirconium oxides for the selective purification of solutions containing radioactive iodine. Currently, he works as a senior inspector at the Finnish Radiation Safety Authority (STUK) and his work focuses on the radiochemical and ICP-MS analytics of radionuclides.

Miho Otaki earned her Bachelor’s degree in chemistry from the University of Tokyo and pursued her Master’s degree in radiochemistry at the University of Helsinki. Her Master’s research involved the synthesis and characterization of ion-exchange sorbents for the removal of radioactive substances. Currently, she is a doctoral researcher at the University of Helsinki, working with Dr Risto Koivula. Her ongoing project is focused on developing highly selective methods for the separation of Group 3 and f-block elements, utilising inorganic–organic hybrid sorbents and selective precipitation techniques.

Topi Suominen is a PhD researcher at the University of Helsinki, working in the ion exchange group of the radiochemistry laboratory. He earned his Master’s degree in chemistry and molecular sciences from the University of Helsinki in 2019. Originally focusing on environmental chemistry during his Master’s, he moved into materials research in the ion exchange group under the supervision of Risto Koivula in 2020 when he started his PhD. Currently, his research interests include separation of lanthanides and synthesis of mesoporous metal oxides.

## Inorganic Chemistry

**Anderson Moledo Vicente Guedes, Federal University of Rio de Janeiro, Brazil** 
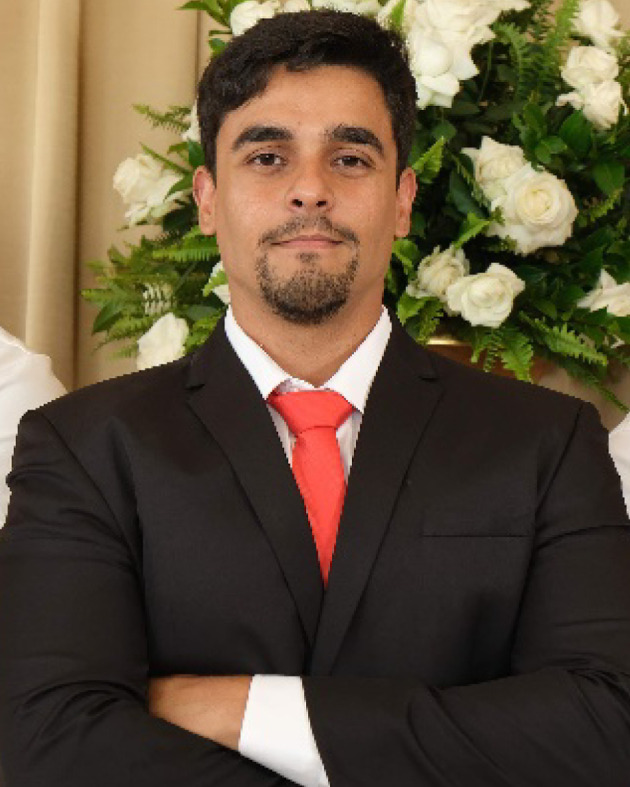


Anderson is recognised for his outstanding contribution to the research presented in:

*Valence tautomerism in a cobalt–dioxolene complex containing an imidazolic ancillary ligand* (https://doi.org/10.1039/D3RA03235C)

Anderson Moledo Vicente Guedes attended chemical engineering college and obtained a Bachelor’s degree in 2016. Later in 2016, he entered the Master’s course in chemistry at the Federal University of Rio de Janeiro, Brazil, under the supervision of Prof. Giordano Poneti and Prof. Rafael Alves Allão Cassaro. Here, he worked on the synthesis and the magnetic properties of transition metal complexes seeking to obtain the phenomenon of valence tautomerism. After completing his Master’s degree in 2018, he joined the doctorate course in 2019 at the same University. During his doctorate, Anderson worked to increase the complexity of systems that exhibit the phenomenon of valence tautomerism and used various techniques to study the complexes he developed, having contact with techniques such as synchrotron X-ray absorption spectroscopy, X-ray photoelectron spectroscopy, magnetometry and others. Anderson is currently in the process of completing his doctorate and, concomitantly, using part of the knowledge acquired during postgraduate studies in a profession as a forensic expert.

## Materials Chemistry

**Kun-Lin Wu, University of Washington (UW), USA** 
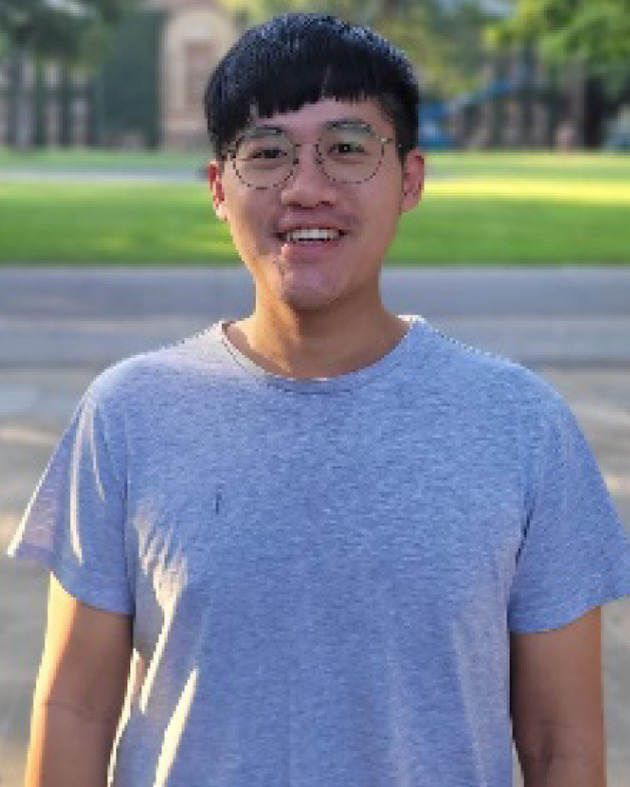


Kun-Lin is recognised for his outstanding contribution to the research presented in:

*Pharmacological regulation of protein-polymer hydrogel stiffness* (https://doi.org/10.1039/D3RA04046A)

Kun-Lin Wu is a PhD candidate in chemical engineering at the University of California, Davis, under the direction of Prof. Ambarish Kulkarni. His current research interest is using computational modelling to investigate CO_2_ adsorption in microporous materials for carbon reduction application. Before his PhD studies, he was a Master’s student at the University of Washington under the guidance of Prof. Cole DeForest. He employed protein engineering and molecular biology techniques to design and synthesise biomaterials for drug delivery application. His research interest is developing and designing materials from an atomistic scale with applications in diverse domains, including carbon capture, nanoscience and biomaterials.

## Nanoscience

**Ashima Makhija, Maharshi Dayanand University, India** 
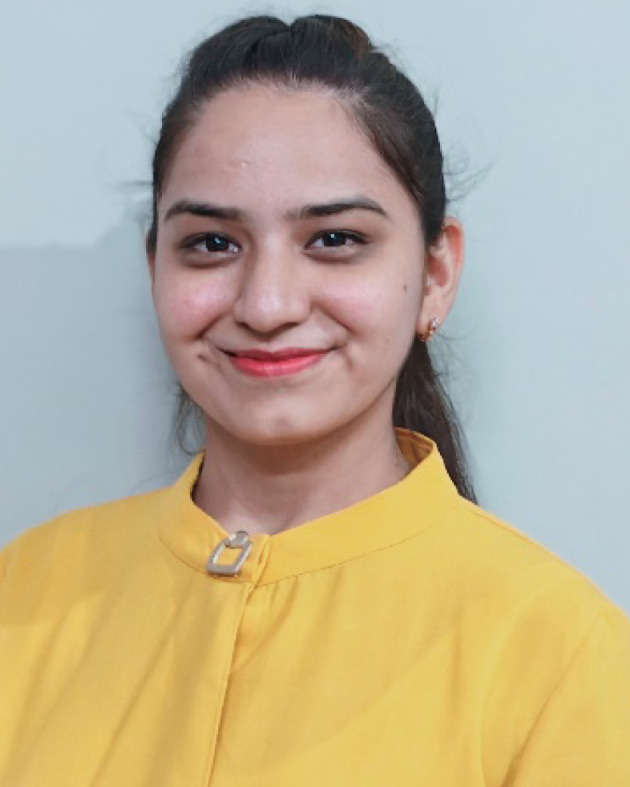


Ashima is recognised for her outstanding contribution to the research presented in:

*Green emission from trivalent cerium doped LaAlO*_*3*_ */MgO nano-composite for photonic and latent finger printing applications* (https://doi.org/10.1039/D3RA02078A)

Ashima Makhija is a research scholar in the Department of Physics at Maharishi Dayanand University, India. Currently, she is pursuing a PhD on the topic “Synthesis and Characterization of Rare-earth metal doped nanomaterials”. Ashima’s research interest lies in enhancing luminescence and the photocatalytic properties of nanomaterials, contributing to advancements in Materials Science and Nanotechnology. Under the expert guidance of Prof. Rajesh Punia, she embarked on a journey to explore the suitability of luminescent materials for LEDs and latent fingerprint identification applications. Her research work has led to the publication of seven research papers in peer-reviewed international journals and one in conference proceedings. Ashima has actively participated in numerous international and national conferences, workshops, seminars, and webinars that have enriched her research experience. She has received the Best Poster Presentation Awards at 2 National Conferences, *i.e.* RAMAN-2023 and FMS-2024. Her academic achievements include securing 3rd rank in graduation at the university and qualifying for GATE 2018. She has also been awarded the POSE fellowship at graduation and post-graduation levels.

## Organic Chemistry

**Margarita Damai, London Metropolitan University, UK** 
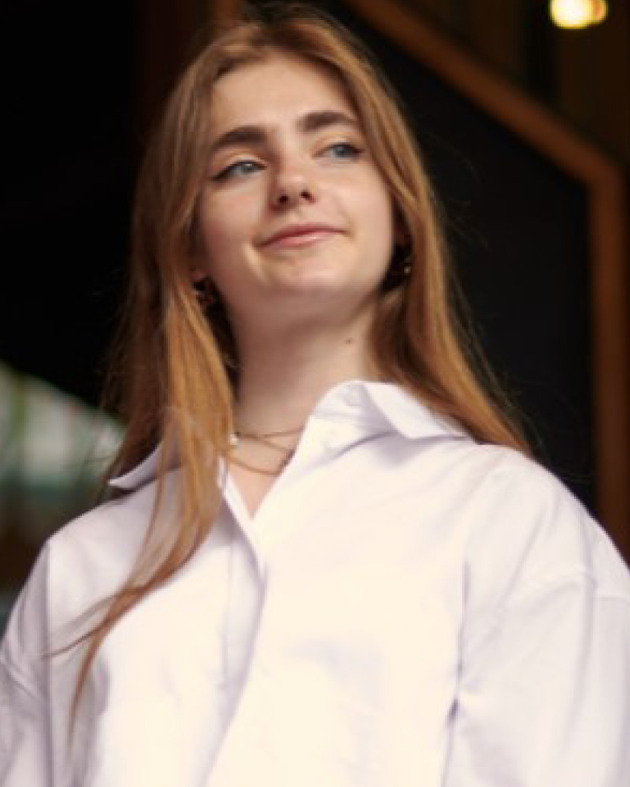


Margarita is recognised for her outstanding contribution to the research presented in:

*Crafting mono- and novel bis-methylated pyrroloquinoxaline derivatives from a shared precursor and its application in the total synthesis of marinoquinoline A* (https://doi.org/10.1039/D3RA05952A)

Margarita Damai is a second-year student at London Metropolitan University pursuing a BSc biomedical sciences degree. Her research work primarily revolves around synthesising complex molecular structures and their applications in medicinal chemistry.

She is an active individual and has participated in several research conferences to represent her work. Apart from research, Margarita is heavily involved in democracy, particularly within the LondonMet Students Union, where she served one year as a sabbatical officer.

## Physical Chemistry

**Maria Dekermenjian, INRS-EMT, Canada** 
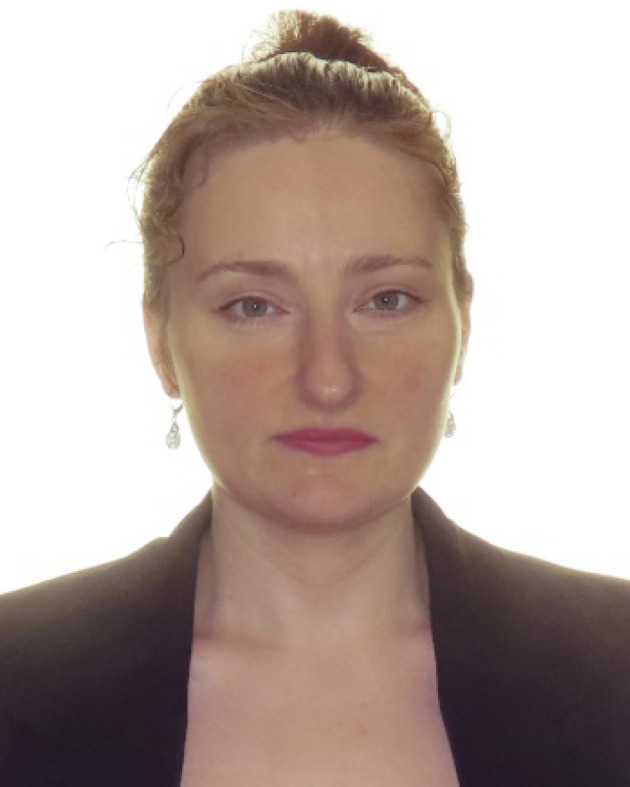


Maria is recognised for her outstanding contribution to the research presented in:

*Raman spectroscopy investigation of* magnesium *oxide nanoparticles* (https://doi.org/10.1039/D3RA04492K)

Since high school, Maria Dekermenjian has found an interest and passion in science, especially in mathematics and physics. She completed her Bachelor’s degree in physics at the University of Montreal (2005–2008). Her real contact with research was during the three summer internships (financed by NSERC and RQMP scholarships) with her physics professors during her Bachelor’s degree. She wanted to learn more about research, so she pursued a Master’s degree in physics in the group of Prof. Richard Martel at the same university from 2008 to 2011. Her thesis focussed on far infrared properties of carbon nanotube films. After finishing her Master’s thesis, she worked as a private tutor as well as immersing herself in entrepreneurship. She wanted to learn more about nanotechnology and more importantly perfecting her research skills. Therefore, in 2017, she started a PhD at EMT-INRS (Varennes, Canada) in the group of Prof. Andreas Ruediger co-supervised by Prof. Alexandre Merlen. Maria graduated in 2024, her thesis being entitled “Raman spectroscopy study of magnesium oxide nanoparticles”. She co-supervises research activities in the same field. Thus far, she has 10 publications in highly impactful journals.

Please join us in congratulating all of our winners for their exceptional achievement. We extend our sincere gratitude to all the authors for their contributions, as well as to the editors and referees for their collaboration, which has resulted in this high-quality series.

We will continue to recognise outstanding student contributions and plan to give out these awards each year. If you published a research article in 2024, or go on to publish with the journal in the future, and would like to recognise a significant contribution made by a student, we invite them to join us in future editions of this series. Please email advances-rsc@rsc.org for more information.

Dr Laura Fisher, Executive Editor for *RSC Advances*

